# Effects of spinal manipulative therapy on inflammatory mediators in patients with non-specific low back pain: a non-randomized controlled clinical trial

**DOI:** 10.1186/s12998-020-00357-y

**Published:** 2021-01-08

**Authors:** Julita A. Teodorczyk-Injeyan, John J. Triano, Robert Gringmuth, Christopher DeGraauw, Adrian Chow, H. Stephen Injeyan

**Affiliations:** 1 Graduate Education and Research Programs, Canadian Memorial Chiropractic College, Toronto, Ontario Canada; 2 Division of Clinical Education, Canadian Memorial Chiropractic College, Toronto, Ontario Canada; 3 Private practice, Richmond Hill, Ontario Canada; 4 Research and Clinical Education, Canadian Memorial Chiropractic College, Toronto, Ontario M2H 3J1 Canada

**Keywords:** Low back pain, Spinal manipulation, Inflammatory mediators, Cytokine

## Abstract

**Background:**

The inflammatory profiles of patients with acute and chronic nonspecific low back pain (LBP) patients are distinct. Spinal manipulative therapy (SMT) has been shown to modulate the production of nociceptive chemokines differently in these patient cohorts. The present study further investigates the effect(s) of SMT on other inflammatory mediators in the same LBP patient cohorts.

**Methods:**

Acute (*n* = 22) and chronic (*n* = 25) LBP patients with minimum pain scores of 3 on a 10-point numeric scale, and asymptomatic controls (*n* = 24) were recruited according to stringent exclusion criteria. Blood samples were obtained at baseline and after 2 weeks during which patients received 6 SMTs in the lumbar or lumbosacral region. The in vitro production of tumor necrosis factor (TNFα), interleukin-1 β (IL-1β), IL-6, IL-2, interferon ɣ (IFNɣ), IL-1 receptor antagonist (IL-1RA), TNF soluble receptor type 2 (sTNFR2) and IL-10 was determined by specific immunoassays. Parametric as well as non-parametric statistics (PAST 3.18 beta software) was used to determine significance of differences between and within study groups prior and post-SMT. Effect size (ES) estimates were obtained using Cohen’s *d.*

**Results:**

Compared with asymptomatic controls, SMT-related change scores were significant (*P* = 0.03–0.01) in reducing the production levels of TNFα in both patient cohorts and those of IL-6, IFNɣ and sTNFR2 (*P* = 0.001–0.02) in patients with chronic LBP. Above-moderate to large ES (*d* > 0.6–1.4) was observed for these mediators. Compared with respective baselines, a significant post-SMT reduction (*P* = 0.01) of IL-6 production was detected only in patients with chronic LBP while a significant increase of IL-2 production (*P* = 0.001 vs. control, and *P* = 0.004 vs. chronic LBP group) and a large ES (*d =* 0.87) were observed in patients with acute LBP. Pain and disability scores declined significantly (*P* < 0.001) in all LBP patients, and were positively correlated (*P* = 0.03) with IFNɣ and IL-2 levels in the acute LBP cohort.

**Conclusion:**

The short course of SMT treatments of non-specific LBP patients resulted in significant albeit limited and diverse alterations in the production of several of the mediators investigated in this study. This exploratory study highlights the potential of SMT to modulate the production of inflammatory components in acute and chronic non-specific LBP patients and suggests a need for further, randomized controlled clinical trials in this area.

**Trial registration:**

This study was prospectively registered April 2012 with Clinical Trials.gov (#NCT01766141).

https://register.clinicaltrials.gov/prs/app/action/SelectProtocol?sid=S0003ZIL&selectaction=Edit&uid=U0001V74&ts=2&cx=-axvqtg

## Background

The use of spinal manipulative therapy (SMT) has been recognized as an effective form of non-pharmacological treatment of non-specific low back pain (LBP) [1–3]. Biomechanical and neurophysiological consequences of SMT have been explored in several studies [4–8]. However, there continues to be a need to examine the cellular and molecular mechanisms of SMT-related effects with a view of enriching the basic science background for further studies in the clinical arena.

Non-specific LBP is the most common form of LBP. The prevalence, etiological factors that may be contributing to its development, and the effectiveness of different modalities for treatment, have been widely discussed and documented [1, 9, 10].

Elevated systemic levels of classical inflammatory mediators such as C-reactive protein, and cytokines including tumor necrosis factor α (TNFα), interleukin 1 (IL-1) and IL-6 have been reported in patients with LBP [11–14] suggesting that spinal pain may encompass inflammatory components. A recent systematic review presented an overview of pro-inflammatory markers in LBP [15]. In the context of SMT several studies have explored its effects on inflammatory aspects of the immune response in asymptomatic subjects [16, 17]. However, studies investigating the relationship between SMT and inflammatory parameters in LBP patients have been limited. To our knowledge, only one study has explored the relationship between SMT and the production of nociceptive/chemotactic cytokines in acute and chronic low back patients utilizing an in vitro model [18]. Pain scores in SMT-treated patients with acute and chronic LBP were associated with a significant reduction of the nociceptive chemokine, macrophage inflammatory protein 1α (CC chemokine ligand, CCL3) levels. On the other hand, SMT-related attenuation of the production of macrophage inflammatory protein-1β, CCL4, was apparent only in patients with acute LBP [18]. Indeed, further studies have demonstrated that inflammatory profiles in LBP patients are quite distinct in acute and chronic cohorts [19]. It was therefore of interest to investigate, in the same patient cohorts, whether SMT effects might differ with respect to the production of other nociceptive/inflammatory mediators.

The present study examined the effects of a series of SMT treatments on inflammatory profiles of patients with acute and chronic LBP. Specifically, the capacity for the production of pro-inflammatory cytokines, TNFα, interleukin 1β (IL-1β), IL-6, IL-2 and interferon ɣ (IFNɣ), as well as anti-inflammatory cytokines IL-10, IL-1 receptor antagonist (IL-1RA) and the anti-inflammatory mediator, soluble TNF receptor type 2 (sTNFR2), were assessed pre- and post-SMT and compared to values from asymptomatic controls. Data on pain intensity pre- and post-SMT were also collected to assess any possible relationships with inflammatory cytokine levels.

## Material and methods

### Trial design

This was a non-randomized, controlled, pre-post SMT intervention trial to explore inflammatory biomarker levels in patients with non-specific acute and chronic LBP, using an in vitro culture model and extending our previous investigations in this context [18, 19]. A cohort of asymptomatic subjects served as control for the confounders of venipuncture and possible temporal changes. Although SMT is the primary variable, venipuncture and temporal factors are additional variables which could affect outcomes. Asymptomatic controls would control for differences in the ability of cells from SMT-treated LBP patients, to produce inflammatory mediators relative to cells from asymptomatic subjects, allowing for comparison of differences in change scores between study groups. Laboratory personnel and data analysts were blinded to the identity and grouping of study participants.

### Participants

Prospective participants presenting to CMCC’s outpatient clinics, between the years 2013 and 2017, were identified consecutively through initial screening and were asked to complete all clinic intake forms including an Oswestry Disability Index (ODI) [20] and a 10-point visual analogue scale (VAS) for pain intensity (Table 1) at presentation. Inclusion criteria were age 22–60 years and having a pain intensity level of 3 or higher on the 0–10 VAS. Exclusion criteria were pregnancy, having received manual treatment of any kind in the preceding 15 days, taken anti-inflammatory medications in the preceding 48 h, reporting any type of unresolved known inflammatory diseases and infections, cancers, coagulopathies, psychological disorders and musculoskeletal conditions other than the presenting LBP condition. Patients were instructed to abstain from anti-inflammatory medications throughout the study period. Finally, refusal to sign the study consent form, or inability to adhere to study schedule also excluded participants.

**Table 1:** Demographic characteristics and measures of pain and disability of subjects enrolled in the study

LBP Patients			
Characteristics	Acute (*N* = 22)	Chronic (*N* = 25)	Asymptomatic Controls (*N* = 24)
Age ± SD	32.8 ± 9.2	36.5 ± 11.1	35.2 ± 10.4
Gender: M/F	13/9	14/11	15/9
VAS 1 ± SD	6.1 ± 1.6	5.2 ± 1.8	NA
VAS 2 ± SD	2.6 ± 1.9 *	2.8 ± 1.9 *	NA
ODI 1 ± SD	37.0 ± 13.7	28.1 ± 8.6	NA
ODI 2 ± SD	14.1 ± 10.3 *	17.3 ± 9.3 *	NA

*P < 0.001; SD: standard deviation; NA: not applicable. VAS 1: 10-point visual analogue scale at admittance; VAS 2: 10-point visual analogue scale post-SMT. ODI 1: Oswestry disability index at admittance; ODI 2: Oswestry disability index post-SMT

**Fig. 1:**
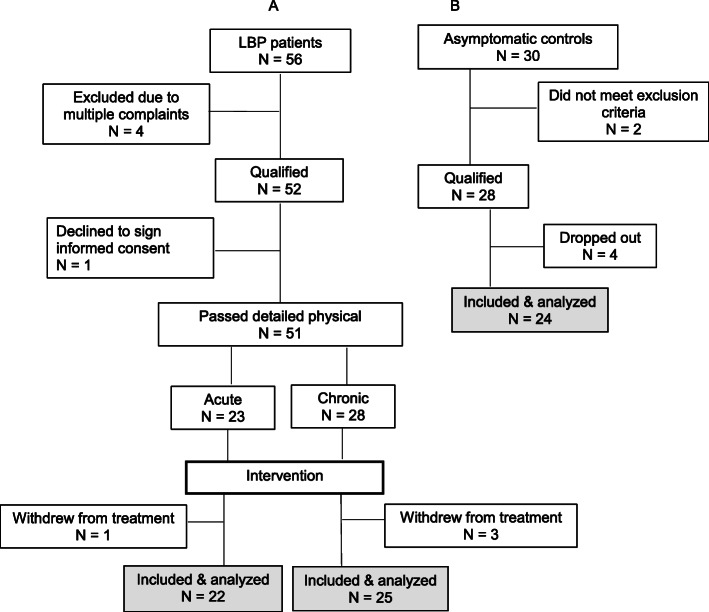
CONSORT diagram showing enrollment and process of exclusion of patients with acute and chronic low back pain (**a**) and healthy asymptomatic control subjects (**b**). (*Modified from Clin J Pain 2019;35:818–825)*

Following the initial screen and meeting the study inclusion/exclusion criteria, 56 LBP patients and 30 asymptomatic age- and gender-matched subjects were recruited (Fig. 1, Table 1). Five of 56 patients were further excluded for different reasons, while 51 underwent full physical examination confirming their non-specific LBP diagnoses (for the purposes of the study LBP was defined as being restricted to the L1- L5 area, with or without sacroiliac joint involvement) and allowing for their designation into acute (*n* = 23) or chronic (*N* = 28) groups (Fig. 1). One acute and 3 chronic LBP patients withdrew from the study. Thus, 22 patients presenting with acute (less than 4 weeks in duration) and 25 with chronic (12 weeks or longer in duration) LBP completed the study. Of the 30 asymptomatic subjects 6 were also excluded reducing the total number of participants in this control group to 24 (Fig. 1). They declared no pain or disability at presentation and were free of LBP for a minimum of one year. In addition, they met all the exclusion criteria for patients. All participants were assigned a numeric code with which to identify their respective intake forms and subsequent blood samples. Thus, all personnel involved in sample and data analysis were blinded.

### Spinal manipulative therapy (SMT)

Immediately prior to the manipulative intervention at the first visit, a baseline blood sample was drawn and taken to the laboratory within one hour (see below). The spinal manipulative intervention consisted of six SMT treatments delivered by the attending clinicians on alternate days in the span of 2 weeks. This protocol was based on the clinical experience of the participating clinicians and published information suggesting symptom control may be achieved as early as after 4–6 SMT treatments [21, 22]. Each treatment (adjustment) involved a single high velocity low amplitude thrust (HVLAT) to the involved segment in the lumbosacral region in the form of a spinal push or spinal pull-type adjustment to the lumbar spine, or a sacroiliac adjustment [23]. Attending clinicians delivered the treatments according to their findings of segmental restrictions in the lumbosacral region on a given day and applied a manipulative thrust to one segment only as indicated by pain or restricted motion upon palpation unlike a typical chiropractic patient encounter when dose and treatment duration are typically longer [22]. Following completion of the six SMT treatments patients were instructed to return 48 h later to provide the post-SMT blood sample and complete the VAS and ODI forms again. Similarly, two weeks after collection of baseline samples a second blood sample was obtained from asymptomatic control subjects, after confirming they had remained free of pain and disability. All blood collections and SMT interventions occurred between 10:00 and 13:00 h.

### Laboratory studies

**Fig. 2:**
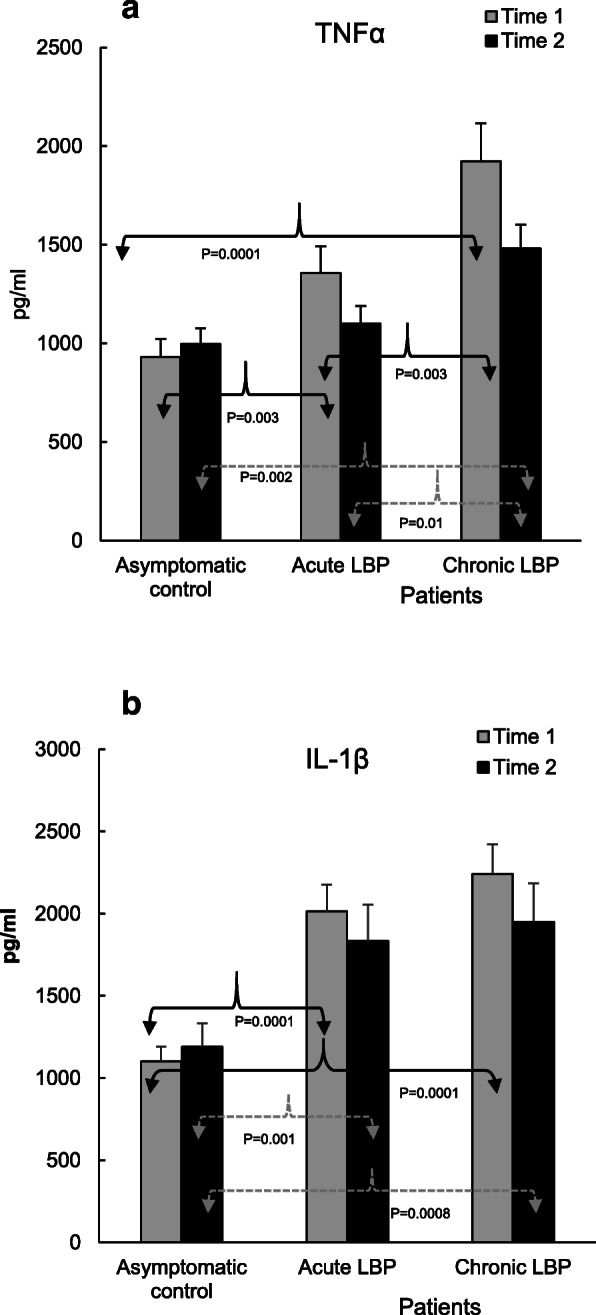
**a**. Production of TNFα in WB cultures from asymptomatic subjects (control) and from patients with acute and chronic LBP determined at baseline (Time 1), and after 2 weeks during which LBP patients received 6 SMT treatments (Time 2). WB cultures were stimulated at initiation with LPS (1 μg/ml) and cultivated for 24 h. Compared with asymptomatic controls, baseline levels of TNFα were significantly elevated both in acute (*P* = 0.003) and chronic LBP patients (*P* = 0.0001) (see ref. [19]). The post-SMT production of TNFα remained significantly (P = 0.002) elevated in patients with chronic LBP. At both study times (baseline and post-SMT), the levels of TNFα in this patient cohort were also significantly higher than those in patients with acute LBP (*P* = 0.003 and *P* = 0.01, respectively). **b**. Production of IL-1β in LPS (1 μg/ml) -stimulated WB cultures from asymptomatic controls and the studied LBP patients. Compared with asymptomatic subjects, the baseline levels of IL-1β production were significantly (*P* = 0.0001) elevated in acute and chronic LBP patients (19) and remained essentially unchanged (P = 0.0008–0.001) following SMT

**Fig. 3:**
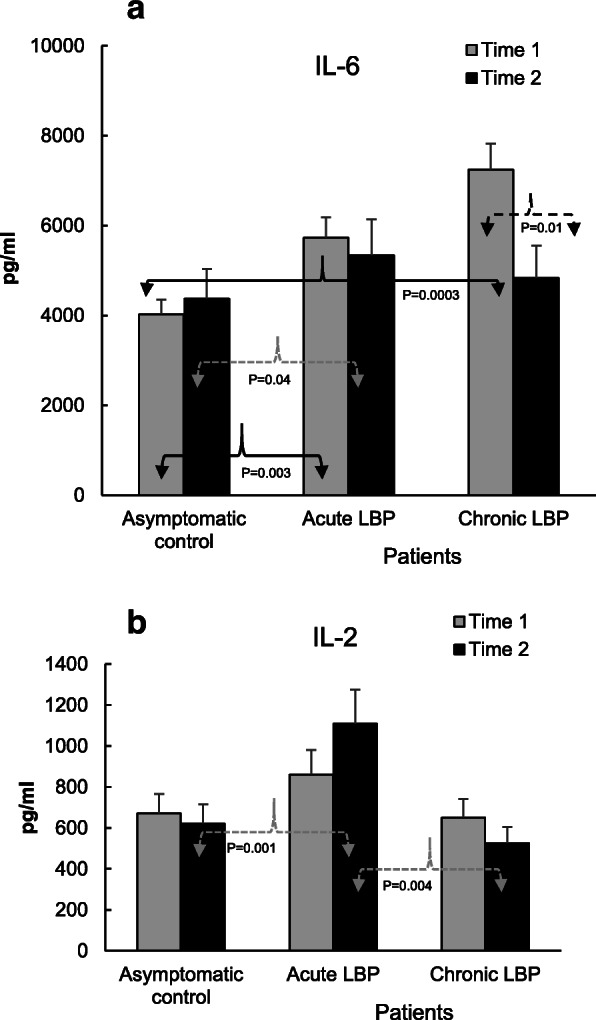
**a**. Effect of SMT on the production of IL-6 in LPS (1 μg/ml)-stimulated WB preparations from patients with acute and chronic LBP. Compared to asymptomatic controls, baseline levels of IL-6 were significantly elevated in both patient groups (*P* = 0.0003–0.003) (19). Post-SMT production of IL-6 declined significantly (*P* = 0.01) in patients with chronic LBP and did not differ significantly from that in asymptomatic controls tested 2 weeks after the initial (baseline) evaluation. **b**. Effect of SMT on the capacity for IL-2 production in WB cultures from patients with acute and chronic LBP. WB preparations from the studied subjects were cultured for 48 h in the presence of phytohemagglutinin (PHA) at a concentration of 10 μg/ml. At baseline, the levels of IL-2 production in both LBP patient groups were comparable with those in asymptomatic subjects (19). Following SMT, the production of IL-2 became significantly elevated in the acute LBP group compared with both asymptomatic controls tested at Time 2 (*P* = 0.001) and patients with chronic LBP (*P* = 0.004)

**Fig. 4:**
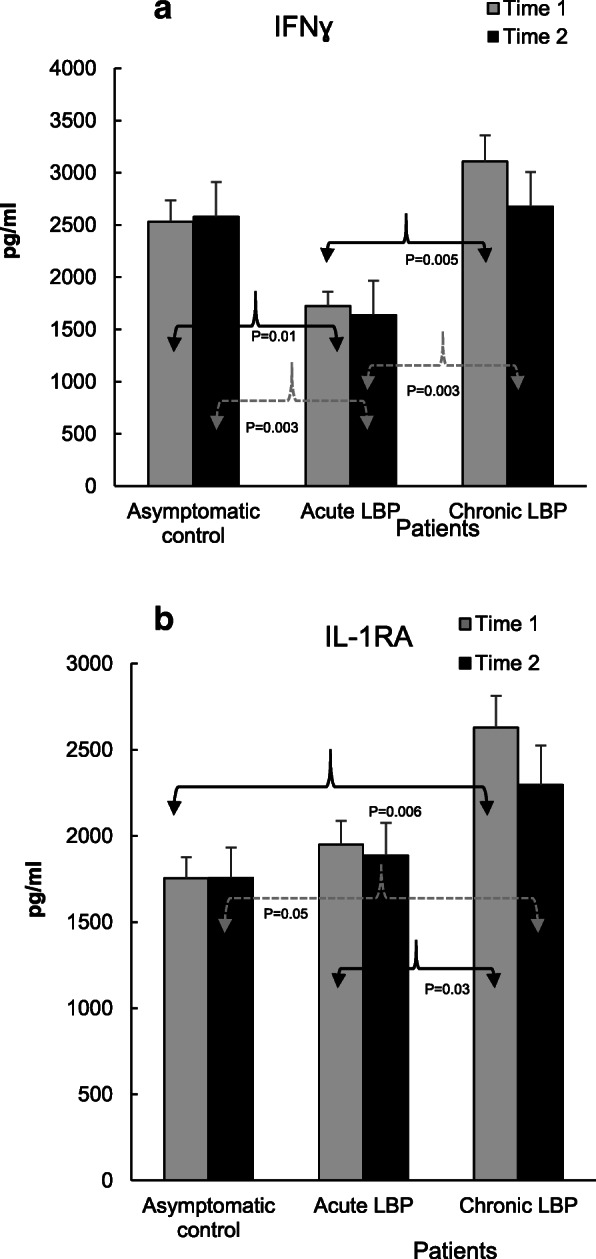
**a**. PHA (10 μg/ml)-induced production of IFNɣ in asymptomatic control subjects and patients with acute and chronic LBP. At baseline, the mean levels of IFNɣ production in patients with acute LBP were significantly reduced in comparison with both asymptomatic controls (P = 0.01) and patients with chronic LBP (*P* = 0.005) (19). The post-SMT production of this cytokine remained significantly lower in acute LBP patients compared to the other study groups. **b**. Production of IL-1RA in WB cultures from the studied subjects. WB preparations from the studied controls and patients with acute and chronic LBP were stimulated with LPS (1 μg/ml) for 24 h. In patients with chronic LBP, the baseline production of IL-1RA was significantly increased in comparison with asymptomatic subjects and patients with acute LBP patients, (*P* = 0.006 and *P* = 0.03, respectively) (19). Following SMT treatments, the mean level of IL-1RA production in this patient group remained significantly higher (P = 0.05) than that in asymptomatic control

**Fig. 5:**
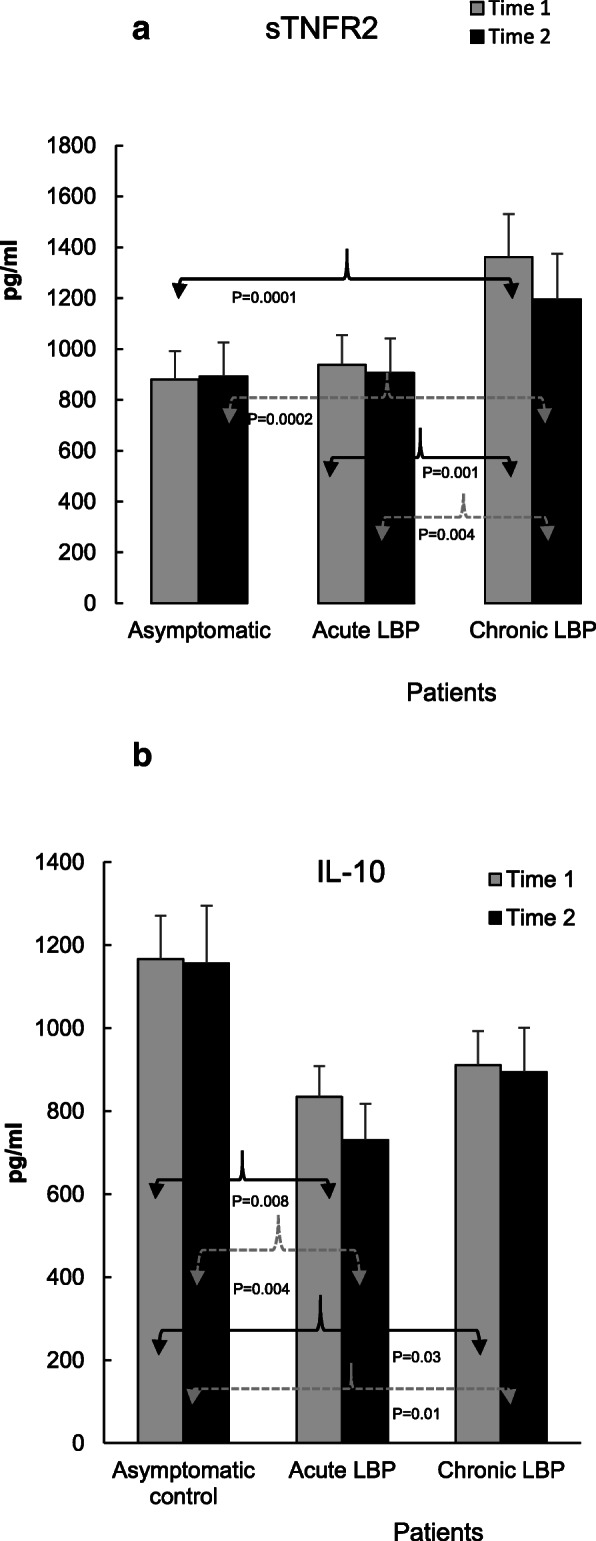
**a**. Production of sTNFR2 in the studied subjects was induced by 48 h stimulation of WB cultures with the combination of LPS and PHA (1 μ/ml and 10 μg/ml, respectively). Baseline levels of sTNFR2 were significantly increased (P = 0.0001–0.001) in patients with chronic LBP, when compared with both asymptomatic subjects and patients with acute LBP (19), and were not altered post-SMT. **b**. Production of IL-10 in asymptomatic control subjects and LBP patients was induced by 48 h stimulation of WB preparations with the combination of LPS and PHA (1 μ/ml and 10 μg/ml, respectively). Compared with the control group, the production of IL-10 was significantly reduced at baseline (19) and was not changed following SMT treatments in patients with acute (as well as chronic LBP patients (*P* = 0.03 and P = 0.01, respectively)

Samples of heparinized peripheral blood (7–8 ml each) from the studied LBP patients and asymptomatic controls were collected by a registered nurse from the antecubital fossa area of the arm. Samples were coded and transferred, at room temperature, to the research laboratory within 60 min of collection and processed immediately for whole blood (WB) culture preparations. Supernatants from inducer-activated cultures were collected, aliquoted and frozen at − 80 C until further analysis. The production of inflammatory mediators (TNFα, IL-1β, IL-6, IL-1RA, IL-2, IFNɣ, sTNFR2 and IL-10) was assessed as described in detail elsewhere [18, 19], and briefly outlined for convenience in captions for Figs. 2, 3, 4, and 5. Specific enzyme-linked immunosorbant assays (DuoSet ELISA development system) for natural and recombinant human cytokines and for natural sTNFR2 (R&D Systems, Minneapolis, MN) were used to quantify mediator levels according to the manufacturer’s recommendations. Mediator concentrations were determined using Gen5 Data Analysis Software (Bio-Tech). Detection limits for TNFα, IL-2 and IFNɣ were 15.6 pg/ml; and for IL-1β, IL-6, sTNFR2, IL-10, IL-1RA - 3.9, 9.4, 12.5, 32 and 39 pg/ml, respectively. Each of the studied samples was tested using R&D kits of the same batch, and assayed a minimum of 3 times at 2–4 different dilutions.

### Sample size

Results published with respect to TNFα levels in patients with chronic neck pain vs. healthy controls [24] were used to calculate a sample size estimate for the present study. Based on a two-tailed t-test of independent groups, using tables provided by Cohen [25] to detect an effect size of 1.00 using alpha = 0.05 and power of 80%, the sample size required was no less than 17 subjects per group. As indicated in Fig. 1, group sizes in the present study were larger.

### Data analyses

The primary outcomes for this study were established as differences in the production of inflammatory mediators between and within the study groups determined at the time of admission into the study (baseline, Time 1) and after the completion of SMT treatments or the second blood sampling for the control group (Time 2). Statistical analyses of data were carried out using PAST 3.18 beta software [26]. Data obtained at both study times were tested for normality using the Shapiro Wilk test. Where non-normal distributions were found, data was transformed (Box-Cox) and analyses repeated. Where tests for equal variances failed, Kruskal-Wallis test was used to confirm results. Testing for differences between the study groups was performed using *t* test for independent samples. Assessment of differences within control and LBP patient groups was carried out by a paired *t* test. One-way ANOVA was used to assess significance of between-group differences in change scores between time 1 and time 2, that is between baseline and the second assessment values in the control, and the baseline and post-intervention values for LBP patients. Cohen’s *d/SD*_*pooled*_ [25] was used to obtain an estimate of effect size (ES) and was interpreted using the benchmarks of small (0.2), moderate (0.5) and large (0.8). ES was assessed using the means of between-groups difference scores. Pre- vs post- SMT values of VAS and ODI (secondary outcomes) were analyzed using a paired *t* test. Spearman correlation coefficients and their statistical significance were also determined to assess the relationship between self-reported pain scores and levels of inflammatory mediator production in patients with acute and chronic LBP. The results depicted in Figs. 2, 3, 4 and 5 and in the text are shown as means ± SEM*. P* values ≤0.05 were accepted as being significant.

## Results

Twenty-two acute and 25 chronic LBP patients as well as 24 asymptomatic subjects completed the study. As reported previously, the demographic characteristics of all participants were comparable. Also, baseline pain and functional scores were comparable between acute and chronic LBP groups [19]. Following the application of six SMT treatments within the span of 2 weeks these scores declined significantly (*P* < 0.001) in both groups of LBP patients (Table 1).

Figures 2, 3, 4 and 5 illustrate the means of values of mediators determined two-weeks apart (Time 1 vs Time 2) for asymptomatic controls, and at baseline (i.e. pre-SMT, Time 1) as well as post-SMT (Time 2) mean values for acute and chronic LBP patients. *P* values, shown in Figs. 2, 3, 4 and 5 under brackets pointing to appropriate columns, show the significance of differences between and/or within the 3 study groups. Comparisons between the study groups at baseline have been carried out previously [19] and are indicated in Figs. 2, 3, 4 and 5 for convenience. Within group i.e. pre- vs. post-SMT comparisons, yielded significance only for IL-6 in the chronic LBP cohort (Fig. 3a). Within group differences for all other mediators in both patient groups were not significant and are not shown. SMT-associated differences in change scores in production levels of the studied mediators and SMT-related effect sizes are shown in Table 2.

**Table 2:** Significance of differences (P) in change scores and SMT-related effect size (*d*) in inflammatory mediator production between the baseline and 2 weeks later, during which patients with LBP received six SMT treatments

Mediator^a^	Within group percent change from baseline (%Δ) Between group Cohen’s effect size *(d)* Significance of differences (P) in change scores		
	Acute LBP	Chronic LBP	Chronic LBP vs. Acute LBP
**TNFα**	%Δ = −17	%Δ = − 22	
	***d = 0.66***	***d = 0.69***	*d = 0.19*
	***P*** **= 0.03**	***P*** **= 0.02**	*P* = 0.53
IL-1	%Δ = −10	%Δ = − 9	
	*d = 0.38*	***d = 0.74***	*d = 0.31*
	*P* = 0.18	*P* = 0.15	*P* = 0.40
**IL-6**	%Δ = − 8	%Δ = − 33	
	*d = 0.5*	***d = 1.45***	***d = 0.64***
	*P* = 0.06	***P*** **= 0.001**	***P*** **= 0.03**
IL-2	%Δ = + 31	%Δ = − 10	
	***d = 0.87***	*d = 0.29*	***d = 0.62***
	***P*** **= 0.02**	*P* = 0.23	*P* = 0.09
**IFNɣ**	%Δ = − 2	%Δ = − 10	
	*d = 0.17*	***d = 0.66***	***d = 0.62***
	*P* = 0.64	***P*** **= 0.02**	*P* = 0.18
IL-1RA	%Δ = − 1	%Δ = − 12	
	*d = 0.28*	*d = 0.3*	d = *0.19*
	*P* = 0.79	*P* = 0.17	*P* = 0.33
**sTNFR2**	%Δ = -3	%Δ = −11	
	*d = 0.18*	***d = 0.67***	*d = 0.19*
	*P* = 0.37	***P*** **= 0.01**	P = 0.09
IL-10	%Δ = −12	%Δ = − 11	
	*d = 0.15*	*d = 0.36*	*d = 0.48*
	*P* = 0.39	P = 0.2	*P = 0.053*

^a^Mediators for which statistical significance and above moderate effect size were found are shown in bold. Direction of changes is indicated by “- “or “+” signs

Relative to their respective baselines, post-SMT levels of TNFα were reduced in both LBP patient groups (%Δ 17–22, Table 2) but did not reach statistical significance (Fig. 2a). In patients with chronic LBP, post-SMT levels of TNFα remained significantly elevated compared with both the control (*P* = 0.002) and the acute LPB (*P* = 0.01) groups (Fig. 2a). Compared with asymptomatic controls, differences in change scores in TNFα production were significant in patients with both acute (*P* = 0.03) and chronic LBP (*P* = 0.02) (Table 2) and the SMT related ES was moderate (d = 0.66–0.69).

The post-SMT production of IL-1β relative to baseline remained essentially unchanged and its levels remained significantly elevated (*P* = 0.0008–0.001) in both patient groups when compared with asymptomatic subjects tested at Time 2 (Fig. 2b). There was no change score difference in either LBP cohort (Table 2). However, ES was above moderate (*d =* 0.74) in the chronic LBP cohort.

In the acute LBP cohort, SMT treatments had no significant effect on the production levels of IL-6 relative to baseline and remained elevated (*P* = 0.04) relative to asymptomatic controls (Fig. 3a). However, in chronic LBP patients the post-SMT production of IL-6 decreased (%Δ = − 33, Table 2) and was significantly reduced (P = 0.01) compared to baseline (Fig. 3a). Differences in change scores in the level of IL-6 production between control and chronic as well as acute and chronic LBP patients were statistically significant (*P* = 0.001 and P = 0.03, respectively, Table 2) and SMT-related ES for the chronic patient cohort was large (*d* = 1.45, Table 2).

Following SMT, the levels of IL-2 in acute LBP patients increased (%Δ = + 31, Table 2) and became significantly higher compared to those in asymptomatic controls (P = 0.001) and to patients with chronic LBP (*P* = 0.004; Fig. 3b). Difference in change score for this cytokine was significant (*P* = 0.02) and treatment ES (*d* = 0.87) was large (Table 2).

The level of production of IFNɣ in the acute LBP patient group, significantly reduced at baseline, was not altered following SMT (*P* = 0.003; Fig. 4a). Also, in patients with chronic LBP, post-SMT production of this cytokine did not change significantly (Fig. 4a). However, difference in change scores in the production levels of IFNɣ in the chronic cohort was significant and treatment ES was moderate (P = 0.02, *d* = 0.66, Table 2).

In patients with acute LBP, self–assessed pain scores and baseline levels of IFNɣ production have been shown to be positively correlated [19]. This was also observed in the present study following SMT along with a positive correlation between VAS scores and IL-2 production levels (Table 3). No such correlation between levels of inflammatory mediators and pain scores were apparent in SMT-treated patients with chronic LBP.

**Table 3:** Relationship between visual analogue (VAS 2) scores and post-SMT levels of pro-inflammatory cytokine production in patients with acute and chronic LBP

Cytokine	Acute LBP		Chronic LBP	
	r_s_	Significance (P)	r_s_	Significance (P)
TNFα	0.05	NS	0.13	NS
IL-1β	0.30	NS	0.17	NS
IL-6	0.06	NS	0.18	NS
**IL-2**	0.47	**0.03**	0.09	NS
**IFNɣ**	0.44	**0.03**	−0.26	NS

Spearman correlation coefficients (r_s_) and their statistical significance (P values) were calculated to assess the relationship between VAS 2 pain scores and post-SMT inflammatory mediator production levels in patients with acute and chronic LBP

*NS* not significant

With respect to anti-inflammatory mediators, the baseline production of IL-1RA and sTNFR2, was significantly augmented in patients with chronic LBP (*P* = 0.03 and 0.0001, Figs. 4b and Figs. 5a respectively). Following SMT, the production of IL-1RA in this group decreased somewhat but remained, nonetheless, significantly higher (*P* = 0.05) than that in the asymptomatic control group (Fig. 4b). Similarly, the post-SMT levels of sTNFR2 in patients with chronic LBP remained significantly higher than those in the control (*P* = 0.0002) and in patients with acute LBP (*P* = 0.004, Fig. 5a). Nonetheless, SMT-related change scores in the production of sTNFR2 in the chronic LBP group were significantly different from those in asymptomatic controls and ES was moderate (*P* = 0.01, *d* = 0.67, Table 2).

Relative to controls, post-SMT levels of IL-10 stayed significantly reduced, both in acute (P = 0.004) and chronic (P = 0.01) patient cohorts.

## Discussion

In concordance with our previous report [19], Figs. 2, 3, 4, 5 demonstrate that, baseline capacity for the inducible production of inflammatory mediators differs significantly between the acute and chronic LBP patient cohorts and in comparison, to asymptomatic controls. Post-SMT values of individual mediators revealed a general trend towards lowering of the production of pro-inflammatory cytokines (TNFα, IL-1β, IFNɣ, Figs. 2 and Fig. 4a). Although, these changes did not achieve statistical significance, determinations of Cohen’s *d* revealed a moderate ES (*d* > 0.5) for reduced TNFα production in both patient cohorts and for all other proinflammatory cytokines, except IL-2, in patients with chronic LBP (Table 2). Large SMT-related ES was observed for enhanced IL-2 production in the acute LBP cohort and in reduced IL-6 production in the chronic LBP group (Table 2). This is consistent with the significant effect of SMT on the production of these cytokines relative to baseline. In patients with acute LBP, a significant increase in post-SMT IL-2 levels was observed compared with both the control and patients with chronic LBP (Fig. 3b). In patients with chronic LBP, the post-SMT production of IL-6 declined significantly compared with baseline though it remained slightly elevated compared to controls (Fig. 3a).

IL-6 is a pleiotropic cytokine recognized as a strong mediator of chronic inflammation [27] involved in the pathogenesis of inflammatory pain [28]. Association of IL-6 with severity of pain in LBP has been reported [15]. Diminished production of IL-6 in the chronic LBP cohort might therefore reduce its nociceptive action [29], although no significant correlation was found between reduced VAS 2 scores and levels of IL-6 production post-SMT in either patient group (Table 3). Importantly, following SMT a decline in the production of the nociceptive chemokine CCL3, the regulation of which is linked to the strength of IL-6 trans-signaling [30] has also been observed in the chronic LBP patient cohort [18]. The SMT-associated decrease of IL-6 levels could possibly modify the classic anti-inflammatory pathway of IL-6 signaling via its membrane-bound receptor, mIL-6R [28]. However, the interaction between IL-6 and mIL-6R has been shown to increase the production of anti-inflammatory mediators such as IL-1RA and sTNFR [31] which was not observed in the present study (Figs. 4b and Fig. 5a). It is also unlikely that attenuation of IL-6 production following SMT could be related to increased activity of anti-inflammatory IL-10 [32] since no significant alterations in the level of IL-10 release were observed in either study group (Fig. 5b). Thus, physiological mechanism(s) and possible clinical consequences of the decline in IL-6 production following SMT in chronic LBP patients warrants further investigation.

In patients with acute LBP, the post-SMT production of CD4 + Th1 lymphocyte–derived cytokine, IL-2, was significantly up-regulated and that of IFNɣ remained significantly reduced compared to both asymptomatic controls and patients with chronic LBP (Figs. 3b and Fig. 4a). IL-2 may act not only as proinflammatory but also immunoregulatory and immunostimulatory mediator [33]. Moreover, at certain concentrations and in combination with other cytokines both IL-2 and IFNɣ may function as anti-inflammatory cytokines [34]. At low concentrations, IFNγ suppresses T cell trafficking to the site of inflammation [35] while IL-2, in combination with other cytokines, may down-regulate T-cell activation [36]. Based on the outcomes of the present study, it cannot be determined if SMT-related alterations in IL-2 production, combined with attenuation of IFNγ levels in patients with acute LBP, might be potentially considered pro-inflammatory or immunomodulatory. A study exploring the systemic (in vivo) interactions of IL-2 with other soluble mediators of inflammation [34] will be needed to clarify the issue. Interestingly, IL-2 has been reported to exert an analgesic effect in an experimental model of neuropathic pain [37]. Thus, elevation of its production in response to SMT may be consistent with hypoalgesic effects of various forms of manual therapy including spinal manipulation [38, 39], and modulation of nociceptive information [6]. Recently, Molina–Ortega et al. [40] described increases in substance P (SP) levels and elevation of pressure pain threshold following cervical manipulation in asymptomatic subjects. Of interest, it has been shown that SP upregulates IL-2 expression in activated human T cells [41].

Our results indicate, SMT did not alter anti-inflammatory mediator production levels (IL-1RA, IL-10 Figs. 4b, and Fig. 5b) in a direction to produce a physiological counterbalance to the reduced pro-inflammatory mediator levels (Figs. 2, 3, 4a). In fact, a significant reduction in change scores, associated with moderate effect size in attenuation of sTNFR2, was observed in the chronic LBP cohort (Table 2, Fig. 5a). Our previous study [19] had shown that compared with asymptomatic group, the ratios of TNFα, IL-1β, IL-6 and IL-2 to IL-10 levels at baseline were significantly elevated in both LBP patient groups. The determinations of the same ratios post SMT, in the current study, showed no significant alterations in their values (not shown) suggesting a sustained imbalance between proinflammatory and anti-inflammatory mediator levels favoring the production of proinflammatory components. Physiological mechanisms of inflammation and pain control may be mediated by factors beyond conventional humoral anti-inflammatory conduits [42]. Regulation of the inflammatory response through reflex mechanisms operating via autonomic circuits has been investigated [43, 44]. Thus, it could be hypothesized, that SMT may provide sufficient afferent stimulus to autonomic nervous system and provoke an anti-inflammatory reflex modulating the response of inflammatory cells. Further studies are necessary to explore the cellular/molecular mechanisms of SMT effects on inflammatory response in LBP, including the assessment of potential quantitative changes within the population of PBMCs. A phenotypic study of PBMCs from patients with acute and chronic LBP is currently underway in our laboratory to address this issue.

The short course of SMT applied in this study did not result in complete resolution of clinical outcomes (VAS and ODI, Table 1), which is consistent with the limited alterations in the production levels of inflammatory mediators studied herein, as well as with limited changes seen in the production of nociceptive CC-series chemokines and endothelial cell activation reported previously [18]. The persistence of inflammatory mediator production in patients with acute LBP may present a significant contributing factor in the pathophysiology of chronic low back pain, which eventually affects close to two thirds of patients reporting initially with acute spinal pain [45].

This study had several limitations. In relation to the protocol of patient selection, we endeavored to exclude patients who did not meet strictly our inclusion criteria for acute or chronic LBP. Although unlikely, some cross-contamination of these cohorts might have occurred inadvertently due to the subjective nature of patient reporting and clinical decision making. As a result, subacute cases might have been allocated into one or the other LBP cohort. More than one clinician was involved in delivering the manipulative interventions, which may have resulted in variability in HVLAT forces applied for the adjustment. Guided by practical and methodological considerations dictated by the design of the study, both the number and duration of the intervention were different from a typical chiropractic treatment plan, which may involve multiple adjustments and repeated visits over a span of several weeks [21]. Furthermore, the study did not include a post-intervention follow up period. The strict exclusion from the study of LBP patients who had concomitant MSK complaints of any type, made recruitment of qualified subjects extremely difficult, contributing to the small sample size, and made it not feasible to include a sham or no-treatment LBP patient group as controls. Finally, our approach to investigating the effects of SMT on a putative local inflammatory lesion in the context of LBP is new and represents an exploratory, hypothesis generating study, looking for large differences. Multiple hypothesis testing with no correction has inflated the probability of a type I error. On the other hand, the targeted sample size was derived based on ability to detect a large effect size of 1, reducing the likelihood of detecting smaller but clinically relevant changes. We trust future randomized clinical trials designed to address these limitations, will help validate the observations reported in the current study, and will contribute to a better understanding of the efficacy of SMT in modulating inflammatory parameters in patients with non-specific acute or chronic LBP.

## Conclusion

Following a short course of SMT treatments overall alterations in the inducible production of inflammatory mediators in acute and chronic non-specific LBP were limited. However, a significant reduction in the production of IL-6 in chronic patients, and an enhanced IL-2 production in acute patients were observed along with reduction in pain and self-reported functional outcomes. Furthermore, the significance and direction of SMT-related change scores as well as the moderate-to-large effect sizes observed for several mediator levels studied indicate the potential of this intervention to impact the inflammatory process in LBP patients. Collectively, these results suggest that full-scale randomized controlled trials are warranted to further explore the effects of SMT on inflammatory processes in LBP patients.

## Data Availability

Datasets used and analyzed in this study are available from the corresponding author on request.

## Abbreviations

SMT: Spinal manipulative therapy
HVLAT: High velocity low amplitude trust
LBP: Nonspecific low back pain
WB: Whole blood
IL: Interleukin
IL-1RA: Interleukin-1 receptor antagonist
TNFα: Tumor necrosis factor alpha
sTNFR2: Soluble tumor necrosis factor receptor type 2
IFNɣ: Interferon gamma
VAS: Visual analogue scale
ODI: Oswestry disability index
LPS: Lipopolysaccharide
PHA: Phytohemagglutinin
ES: Effect size
